# Assessing the Effects of Stocking Density on Turkey Tom Health and Welfare to 16 Weeks of Age

**DOI:** 10.3389/fvets.2018.00213

**Published:** 2018-09-04

**Authors:** Kailyn Beaulac, Karen Schwean-Lardner

**Affiliations:** Department of Animal and Poultry Science, University of Saskatchewan, Saskatoon, SK, Canada

**Keywords:** turkey, stocking density, welfare, feather condition, feather cleanliness, mobility, footpad lesions, heterophil/lymphocyte ratio

## Abstract

Four levels of turkey tom stocking density (SD) (30, 40, 50, 60 kg/m^2^) were evaluated in two 16 week trials (*n* = 2,868 Nicholas Select). Poults were allocated to one of eight independently ventilated rooms per trial (6.71 × 10.06 m) based on final predicted body weight (two replicates per SD per trial). Room temperature, humidity, carbon dioxide and ammonia concentration were recorded throughout the trial; ventilation was adjusted to equalize air quality across treatments. Mobility (gait score, scale 0–5) was recorded at 12 and 16 weeks (20 birds per replicate). Footpad lesion score (scale 0–4), feather condition (scale 1–4), and cleanliness scores (scale 1–4) were recorded at 10 (Trial 2), 12, and 16 weeks of age (20 birds per replicate). Aggressive injury incidence was recorded daily for Trial 2. Stress (heterophil/lymphocyte ratio) was evaluated at 4, 12, and 16 weeks of age (15 birds per replicate). Behavior was recorded and scan sampled (field of view) at 12 (Trial 1), 14, and 16 weeks. Data were analyzed using regression analysis (linear, Proc Reg; quadratic, Proc RSReg) for relationships between the variables and SD. Differences were considered significant when *p* ≤ 0.05. Mobility and footpad lesions were negatively affected by increasing SD (linear) at 16 weeks only. Feather condition and cleanliness decreased linearly as SD increased at week 10, 12, and 16. The incidence of aggressive damage was higher as SD increased during week 4–8 (quadratic). Heterophil/lymphocyte ratios increased linearly at 4 weeks (similar trend at 12 weeks). Behavior was impacted at 12 weeks of age with standing behavior showing a quadratic response, and walking and total disturbance showing a linear decrease as SD increased. Resting, preening, and comfort behaviors increased linearly (14 weeks), while walking and strutting decreased linearly with increasing SD. Finally, at 16 weeks of age resting, standing, walking, feeding, and total disturbance responded quadratically while preening behavior increased linearly with increasing SD. Results suggest that increasing SD negatively impacts bird health and wellbeing through decreased mobility, increased footpad lesions, poorer feather condition and cleanliness, and behavioral changes, but that very low SD (30 kg/m^2^) may result in increased aggressive damage.

## Introduction

Stocking density (SD) can have a large impact on bird health and wellbeing, which has often been documented in broiler chickens. Many of the studies evaluating turkey SD typically evaluate bird performance, however there are few studies that incorporate health and welfare parameters along with performance. Previous research concerning turkey SD has evaluated the effects on feather condition, mobility, footpad lesions, H/L ratios, and behavior, however many of these studies only evaluate one or two of these parameters with production parameters being the primary focus (1–4).

Increasing SD may result in health challenges due to increased stress, alterations in environment, or alterations in group size. Early studies have shown that poorer feather cover is associated with increasing SD (1). Footpad lesions have also been studied in relation to SD, with an increasing incidence of footpad lesions at higher SD (2). The increased incidence of footpad lesions has been shown to increase with high litter moisture (5), which has also been associated with increasing SD (2). Martrenchar et al. also evaluated bird mobility, and found that as SD increased, gait scores became poorer, indicative of poorer mobility (2). The authors hypothesized that this may be due to reduced activity, although it may also relate to the increased incidence of footpad lesions as they have been associated with pain in previous studies (6). In addition to increased litter moisture, litter temperature has also been shown to increase at higher SD (7).

Heterophil/lymphocyte ratios, considered to be a measure of chronic stress, have been evaluated more recently in relation to SD with no effects seen at 7, 12, 16, and 20 weeks of age when birds were housed up to 58 kg/m^2^ based on final predicted body weight (3). Stocking density has previously been shown to increase the H/L ratio in broilers at 49 days of age, indicating SD may increase stress (8). The impact of SD on behavior has been evaluated, indicating mixed results in relation to aggression, where certain studies have shown increases in aggression with decreased space allowance, Buchwalder and Huber-Eicher (9) others have seen increases in feather pecking at low SD (10). In addition, other studies have seen no significant differences in aggression in relation to SD (2, 4). Martrenchar et al. also noted no changes in walking activity with increasing SD (2).

The objectives of this study were to examine the effects of SD on turkey tom health and welfare to 16 weeks of age. It is hypothesized that increasing SD will have negative effects on bird mobility, footpad lesions, and feather condition and cleanliness, as a result of reduced space allowances which may limit bird activity and exercise. Also, increasing density will result in increased stress triggering an increase in the heterophil/lymphocyte ratio. Finally, bird behavior, specifically mobility, and comfort behaviors will be altered as a result of decreased space allowance.

## Materials and methods

### Experimental design

The impact of graded levels of SD in relation to turkey tom health and behavior was evaluated through one experiment, consisting of two blocked trials resulting in a total of four replicates per treatment. Four levels of estimated final room SD (30, 40, 50, and 60 kg/m^2^) were evaluated from placement to 16 weeks of age. The average actual SD achieved at 16 weeks of age was 32.0, 42.6, 53.8, and 62.2 kg/m^2^.

### Birds and housing

Turkey toms were obtained from a commercial hatchery for each trial (*n* = 1,434; strain—Nicholas Select). The poults were toe (three forward facing toes) and infrared beak treated at the hatchery, and then randomly allocated to one of four estimated final SD treatments. Bird numbers were determined using the predicted final body weight at 16 weeks of age (11) with an additional five percent to account for mortality (total of 122, 161, 198, and 236 birds for treatments 30, 40, 50, and 60 kg/m^2^, respectively). Birds were housed in large individual rooms (8 per trial in total; 6.7 m × 10.0 m = 67.5 m^2^), that allowed for separate control of lighting, temperature, and ventilation.

Poults were brooded on wood shavings with a wheat straw base 7–10 cm thick, followed by wheat straw for the remainder of the rearing period. Brooder rings approximately 7.0 m in diameter were used for the first 10 and 11 days for Trials 1 and 2, respectively. *Ad libitum* feed and water were provided throughout the course of the trial. Aluminum tube feeders with a diameter of 36 cm were used for the first 38 days and a diameter of 44 cm for the remaining time. Water was provided using Lubing EasyLine™ pendulum turkey nipple drinkers (Lubing, Cleveland, TN). The number of feeders and nipple drinkers were provided on a per bird basis, allowing feeder and drinker space to be equal regardless of density. Supplemental feeders and drinkers were provided for the first 10 days in both trials. Birds were fed specific quantities of commercially available diets (12). Diet feed amounts were adjusted to account for total mortality the day prior to a diet change.

Lighting was provided via incandescent bulbs and the initial lighting program was 23L:1D and 40 lux. After 2 days, the daylength was decreased by 1 h each day to a final daylength of 18L:6D. Light intensity was gradually reduced to 10 lux by 7 days of age and 15-min dawn and dusk periods were implemented throughout the course of the trial by gradually adjusting light intensity. Rooms were heated via hot water pipes along three walls of the room, with a set point of 29.0°C for the first 7 days. Heat lamps were used as a supplemental heat source and were placed above the brooder ring for the first 14 days in Trial 1 and 19 days in Trial 2. Temperature decreased by approximately 2°C every week to a temperature of 13°C at 91 days. In Trial 2, the initial brooding temperature was adjusted (+0.5°C in week 1 and +1.0°C in week 2), however it was lowered back to the original curve in week 3 (−1.5°C). Temperature and light intensity were further decreased in both trials to decrease the incidence of aggression. Light intensity was decreased to 5 lux at 31 days (Trial 1 and 2) and further decreased to 3 lux at 95 days (Trial 1) and 87 days (Trial 2), while temperature curve was lowered by 2.0°C at 64 days (Trial 1 and 2) reaching a set point of 11.0°C on week 13.

Temperature and humidity data loggers (iButton Hygrochron™ DS1923-F5#; Maxim Integrated; San Jose, CA) were placed at bird level near the entrance of each room and recorded readings hourly throughout the trial. Average weekly temperature was calculated on a per room basis (12). Relative humidity was increased in the first week by providing humidifiers in each room to target 50% RH as recommended by Aviagen (13). Air movement was controlled through a negative-ventilation system, independently in each room. Air quality, including carbon dioxide (via handheld CO_2_ meter CO240; Extech Instruments; Nashua, NH) and ammonia (via ammonia Dräger-Tubes and a handheld pump; Draeger, Inc.; Houston, TX) were monitored biweekly and weekly (respectively) until differences greater than 20% (CO_2_) or 5 ppm (ammonia) were noted across treatments. Once differences were noted, monitoring became more frequent, and carbon dioxide was monitored three times a week and ammonia was monitored biweekly. If differences were noted across treatments, ventilation rates were adjusted in an attempt to equalize air quality across treatments (12).

Additionally, intact square straw bales were provided (one bale per 40 birds) as environmental enrichment. Each room was checked twice daily for mortality and cull birds. At this time, birds with minor aggressive damage were treated by applying a pine tar paste [used in human medicine as it has anti-bacterial properties (14)] to the affected area. In addition, any broken square bales were replaced with the straw from broken bales remaining in the rooms. No additional litter management was performed. Mortality and culls were replaced until day 11 when the brooder rings were removed, to allow for a more accurate prediction of final SD. Finally, if losses greater than 5% occurred, space was blocked in the last 2 weeks of the trial to ensure final SD was met.

### Data collection

#### Health parameters

In both trials, 20 birds per replication were randomly selected and evaluated for gait score, footpad lesion score, feather condition, and feather cleanliness on week 12 and 16. Due to the presence of low levels of footpad lesions at 12 week in Trial 1, footpads were scored an additional time on week 10 in Trial 2. A total of 20 birds per replicate were evaluated at 10 weeks of age for footpad lesion score, feather condition, and feather cleanliness. Subjective gait scores were conducted by two independent scorers and birds were assigned a score of 0–5 using the gait scoring system from Vermette et al. and Garner et al. shown in Table 1 (15, 16). The two scores were then averaged for each bird. Footpad lesion scoring was conducted by one individual by washing the right footpad with a brush and scored on a scale of 0-4 using the system shown in Table 2 (17). Feather condition was evaluated by one individual on four principle areas of the bird (breast, back, wings, and tail). Birds were feather condition scored using a graduated scale of 1–4 [adapted from Davami et al. (18) and Sarica et al. (19)]. A score of 1 indicated no feather cover, 2 indicated more than 50% of the plumage was missing, 3 indicated < 50% of the plumage is missing, and 4 indicated that the bird had full intact plumage. Overall feather cleanliness was also scored by one observer, using a scale of 1–4 adapted by Forkman and Keeling from the broiler scoring system developed by Wilkins et al. on a scale of 1–8 (20, 21). A score of 1 indicated very clean (>75% of the feathers are free from soiling), 2 indicated moderately clean (50–75% of the feathers are free from soiling), 3 indicated moderately dirty (25–50% of the feathers are free from soiling), and 4 indicated very dirty (< 25% of the feathers are free from soiling).

**Table 1:** Broiler gait scoring technique modified for turkeys (15, 16).

**Gait score**	**Degree of impairment**	**Description**
0	None	Smooth, fluid locomotion. The foot is furled while raised
		Straight legs
1	Detectable, unidentifiable abnormality	The bird is unsteady, or wobbles when walking; however, the problem leg is unclear or cannot be identified in the first 20 s of observation. The bird readily runs from the observer in the pen. The foot may remain flat when raised, but the rest of the stride is fluid and appears unimpaired
		Gait appears unstable (shaky or stomping)
2	Identifiable abnormality that has little impact on overall function	The leg producing the gait defect can be identified within 20 s of observation. If a problem leg is identified after 20 s of observed locomotor behavior then the bird is classed as gait score 1. The defect seems to have only a minor impact on biological function. Thus the bird will run from the observer spontaneously or if touched or nudged. The bird will run, walk or remains standing for at least 15 s after the observer in the pen has ceased to move toward or nudge it. Birds in this, and previous, scores are often observed to scratch their face with their feet, indicating little impact on function. (The most common abnormality in this score is for the bird to make short, quick, unsteady steps with one leg, where the foot remains flat during the step)
3	Identifiable abnormality which impairs function	Although the bird will move away from the observer when approached or touched, or nudged, it will not run, and squats within 15 s or less of the observer in the pen ceasing to approach or nudge it. If the bird squats after 15 s have elapsed it is classified as gait score 2
4	Severe impairment of function, but still capable of walking	The bird remains squatting when approached or gently nudged for 5 s. Bird may appear to rise but still rest upon their hocks. A bird which takes longer than 5 s to rise, or which does not rise at all is scored as 4, while a bird that rises in 5 s or less is counted as a 3 (or lower if its gait is good). Nevertheless, the bird can walk when picked up by the observer and placed in a standing position, but squats immediately following one or two steps. (Squatting often involves a characteristic ungainly backwards fall)
		Bird requires wings for balance
5	Complete lameness	The bird cannot walk, and instead may shuffle along on its hocks. It may attempt to stand when approached but is unable to do so and when placed on feet unable to complete a step with one or both legs

**Table 2:** Footpad scoring technique (17).

**Score**	**Description of footpad**
0	No external signs of footpad dermatitis. The skin of the footpad feels soft to the touch and no swelling or necrosis is evident
1	The pad feels harder and denser than a non-affected foot. The central part of the pad is raised, reticulate scales are separated and small black necrotic areas may be present
2	Marked swelling of the footpad. Reticulate scales are black, forming scale shaped necrotic areas. The scales around the outside of the black areas may have turned white. The area of necrosis is < 1 quarter of the total area of the footpad
3	Swelling is evident and the total footpad size is enlarged. Reticulate scales are pronounced, increased in number and separated from each other. The amount of necrosis extends to one half of the footpad
4	As score 3, but with more than half the footpad covered by necrotic cells

The incidence (presence or absence) and location of aggressive damage was recorded daily (back, wing, neck, head, and snood). Skin tears, though not necessarily due to aggression, were included as birds would typically start pecking at them once they occurred. Birds that were mildly affected were treated with pine tar and the incidence recorded. Birds that were moderately impacted and deemed unsuitable to remain on trial, were weighed, removed from the trial, recorded as a cull bird and placed in a hospital pen. Birds that were severely impacted were euthanized, weighed, and recorded as cull birds. The incidence of aggressive damage was calculated for each 4-week interval (0–4, 4–8, 8–12, and 12–16 weeks) as well as overall (0–16 weeks).

Heterophil/lymphocyte ratios (H/L ratios) were assessed as a measure of chronic stress. A total of 15 birds per replicate were randomly selected and blood was collected from the brachial vein into EDTA tubes using a vacutainer on week 4, 8, 12, and 16. Blood smears were prepared the same day blood was collected. Slides were stained using PROTOCOL™ Hema 3™ (Fisher Scientific; Ottawa, Canada) and were stored in a slide box until read. H/L ratios were determined by counting the number of heterophils and lymphocytes within a field of view under 100X oil magnification until a total number of 100 cells was reached (microscope B-290TB; Optika©; Bergamo, Italy).

#### Behavior data

Video recordings of bird activity were taken over a 24 h time period for week 12, 14, and 16 using one ceiling mounted infrared video camera system (Panasonic WV-CF224FX; Panasonic Corporation of North America, Secaucus, NJ). The cameras recorded to a computer system in continuous real-time mode. Field of view observations (22), with approximately one quarter of the room captured, were performed using instantaneous scan sampling of recorded video at 20-min intervals. The number of birds within the field of view performing each behavior was recorded (video playback via Genetec Omnicast Software, Genetec Inc., Montreal, Canada). The behavioral ethogram used is shown in Table 3.

**Table 3:** Behavioral ethogram for turkey toms, as modified from Martrenchar et al. (2) and Vermette et al. (15).

**Behavior**	**Description of behavior**
Feeding	Standing or sitting with head in the feeder
Drinking	Standing or sitting with head in the drinker
Resting	Lying down, not performing any other behavior. May or may not be sleeping
Standing	Standing, not performing any other behavior
Walking	Bird walking or running. Must take 2 or more consecutive steps
Strutting	Standing or walking slowly with feathers erect and breast thrust forward
Fighting	Two or more individuals, where at least one bird is posturing with head back and breast thrust forward. May or may not include one individual running or jumping at the other
Preening	Manipulating own feathers with the beak while standing or resting
Stretching	Extension of the wings and/or legs
Wing flapping	Flapping both wings
Feather ruffle	Full body shake while standing or resting
Environmental pecking	Pecking at walls, feeder tubes (not feed pan), drinker lines (away from the drinker cups), or litter while standing or resting
Feather pecking	Pecking at a pen mate's feathers while standing or resting. The pen mate typically does not move away
Aggressive pecking	Forceful pecking at a pen mate's head, body, or snood while standing or resting. The pen mate typically moves away
Overall disturbance	A bird in a laying posture opens its eyes, lifted its head or moved its body as a result of another bird walking in front of it, on top of it, touching it, or flapping near it
Severe disturbance	A bird in a lying posture stands up as a result of another bird walking in front of it, on top of it, or flapping near it

### Statistical analyses

Data were analyzed using SAS 9.4 (SAS®9.4, Cary, NC) using the room as the experimental unit. The experiment (Trial 1 and Trial 2) was setup as a randomized complete block design, unless only collected in Trial 2 as described above (completely randomized design). Data were checked for normality using Proc UNIVARIATE and log transformed (log +1) when necessary. Regression analyses were performed using Proc Reg (linear) and Proc RSReg (quadratic) to assess the relationship between treatment and dependent variables for gait score, footpad lesion score, feather condition, feather cleanliness, aggressive pecking, H/L ratio, and behavior. Differences were considered significant if *p* ≤ 0.05 and trends were noted when *p* ≤ 0.10.

## Results

### Mobility and footpad lesions

Stocking density did not affect bird mobility (assessed with the use of subjective gait scoring) at 12 weeks of age (Table 4). At 16 weeks however, mobility was poorer as SD increased (*p* = 0.04, linear). The average footpad lesion score and the frequency of footpad lesions are shown in Table 4. The overall footpad lesion scores at week 10 increased in severity with increasing density (*p* = 0.01, linear). Footpad lesion frequency, expressed as the percentage of birds with a lesion of any size (score 1–4), also increased at 10 weeks of age as SD increased (*p* = 0.02, linear). At 12 weeks of age, there were no significant differences noted in birds housed at different densities for the severity or frequency of footpad lesion scores. At 16 weeks of age, the average footpad lesion score increased in a linear manner as SD increased (*p* = 0.02). Density treatment did not impact the frequency of footpad lesions at 16 weeks of age.

**Table 4:** Effect of estimated final stocking density on turkey tom average gait score (scale 0–5) at 12 and 16 weeks of age, average footpad lesion score (scale 0–4) and percentage of footpad lesions present at 10, 12, and 16 weeks of age.

	***n***	**Estimated final stocking density (kg/m**^**2**^**)**				**SEM^a^**	***p*-value (linear)**	***p*-value (quadratic)**	**Regression equation^b^**

		**30**	**40**	**50**	**60**				
**AVERAGE GAIT SCORE**^c^									
Week 12	4	1.05	0.94	1.09	1.08	0.043	0.55	0.59	–
Week 16	4	1.23	1.76	1.66	1.89	0.106	0.04	0.45	Y = 0.02x + 0.07
**AVERAGE FOOTPAD LESION SCORE**^d^									
Week 10^e^	2	0.25	0.55	0.75	1.03	0.124	0.01	0.94	Y = 0.03x − 0.49
Week 12	4	1.13	1.26	1.59	1.66	0.176	0.23	0.93	–
Week 16	4	1.24	1.20	1.60	2.35	0.189	0.02	0.23	Y = 0.04x − 0.09
**PERCENTAGE OF BIRDS WITH FOOTPAD LESIONS (%)**^f^									
Week 10^e^	2	22.50	42.50	47.50	67.50	7.319	0.02	1.00	Y = 1.40x− 18.00
Week 12	4	60.00	68.75	76.25	77.50	5.825	0.26	0.76	–
Week 16	4	67.50	61.25	66.25	85.00	4.743	0.18	0.18	–

a *Standard error of the mean*.

b *Regression considered significant if p ≤ 0.05*.

c *Score 0 is no impairment and score 5 is complete lameness (15, 16)*.

d *Score 0 is no external signs of a lesion and score 4 is greater than 50% of the footpad covered with necrotic cells (17)*.

e *Week 10 footpad score data for trial 2 only*.

f *The percentage of birds scoring 1–4, exhibiting visual signs of a footpad lesion*.

### Feather condition and cleanliness

At 10 weeks of age (Table 5), feather condition was similar for toms across all treatments on the back or the wings, but differences were noted on the tail and breast feathers with mean feather scores decreasing as SD increases (*p* = 0.04, quadratic and *p* < 0.01, linear, respectively). Mean feather condition scores (total score out of 16) decreased as SD increased with mean scores of 13.65, 13.45, 12.70, and 12.43 for treatments 30, 40, 50, and 60 kg/m^2^, respectively (*p* < 0.01, linear).

**Table 5:** Effect of estimated final stocking density on turkey tom average feather condition score (scale 1–4^a^) of four locations: back, wings, tail, and breast and overall body feather condition score (total out of 16) at 10, 12, and 16 weeks of age.

	***n***	**Estimated final stocking density (kg/m**^**2**^**)**				**SEM^b^**	***p*-value (linear)**	***p*-value (quadratic)**	**Regression Equation^c^**

		**30**	**40**	**50**	**60**				
**WEEK 10**^d^									
Back	2	4.00	4.00	4.00	4.00	0	–	–	–
Wing	2	3.93	4.00	3.95	3.90	0.018	0.45	0.09	–
Tail	2	3.23	2.88	2.45	2.45	0.049	< 0.01	0.04	Y = 0.88e^−3^x^2^−0.11x + 5.65
Breast	2	2.50	2.58	2.30	2.08	0.045	< 0.01	0.08	Y = −0.02x + 3.06
Overall^e^	2	13.65	13.45	12.70	12.43	0.203	< 0.01	0.85	Y = − 0.04x + 15.05
**WEEK 12**									
Back	4	4.00	4.00	4.00	3.89	0.010	< 0.01	0.01	Y = −0.28e^−3^x^2^ + 0.02x + 3.59
Wing	4	3.56	3.69	3.50	3.49	0.029	0.11	0.23	–
Tail	4	2.91	2.80	2.54	2.25	0.035	< 0.01	0.18	Y = − 0.02x + 3.64
Breast	4	2.34	2.16	2.16	1.95	0.031	< 0.01	0.76	Y = −0.01x + 2.68
Overall^e^	4	12.81	12.65	12.20	11.58	0.214	0.02	0.55	Y = −0.04x + 14.18
**WEEK 16**									
Back	4	3.98	3.96	3.94	3.85	0.014	< 0.01	0.18	Y = −0.40e^−2^x + 4.11
Wing	4	3.83	3.66	3.59	3.51	0.027	< 0.01	0.41	Y = −0.01x + 4.10
Tail	4	2.94	2.38	2.24	1.95	0.040	< 0.01	0.05	Y = 0.69e^−3^x^2^−0.09x + 5.08
Breast	4	2.10	1.81	1.80	1.86	0.030	0.01	< 0.01	Y = 0.88e^−3^x^2^−0.09x + 3.89
Overall^e^	4	12.84	11.81	11.56	11.18	0.186	0.01	0.16	Y = −0.05x+14.20

a *Score 1 is no feather cover and score 4 is full intact plumage (18, 19)*.

b *Standard error of the mean*.

c *Regression considered significant if p ≤ 0.05*.

d *Week 10 score for trial 2 only*.

e *Sum of four parts: back, wings, tail, breast, scored on a scale of 1–4*.

At 12 weeks of age (Table 5), SD did not impact wing feather condition. Back, tail, and breast feather condition decreased as SD increased (*p* < 0.01, quadratic; *p* < 0.01, linear; *p* < 0.01, linear; respectively). As a result, the mean feather condition score decreased as SD increased with mean values of 12.81, 12.65, 12.20, and 11.58 for treatments 30, 40, 50, and 60 kg/m^2^, respectively (*p* = 0.02, linear).

Feather condition at 16 weeks of age was negatively impacted on all four areas of the body (Table 5). As a result the mean feather condition score decreased in a linear fashion with increasing density as shown in Table 5 (mean values of 12.84, 11.81, 11.56, and 11.18 for treatments 30, 40, 50, and 60 kg/m^2^, respectively; *p* < 0.01).

Feather cleanliness at 10 weeks of age showed a linear decrease as SD increased with mean cleanliness values of 1.18, 1.28, 1.80, and 1.65 for treatments 30, 40, 50, and 60 kg/m^2^, respectively (*p* = 0.01). Mean cleanliness scores at 12 weeks of age also demonstrated that birds become dirtier as SD increases, with mean values of 1.48, 1.91, 2.20, and 2.36 for treatments 30, 40, 50, and 60 kg/m^2^, respectively (*p* < 0.01, linear). Birds at 16 weeks of age demonstrated that birds were more dirty as SD increased, with mean cleanliness scores of 1.45, 2.06, 2.37, and 2.73 for treatments 30, 40, 50, and 60 kg/m^2^ (*p* < 0.01, linear).

### Incidence of aggressive damage

Stocking density did not impact the total percentage of birds treated for aggressive damage (11.48, 9.01, 9.34, and 16.10 for treatments 30, 40, 50 and 60 kg/m^2^, respectively). When looking at specific injuries however, the incidence of skin tears demonstrated a quadratic relationship with SD, with occurrences at 30 (0.41%) and 60 kg/m^2^ (0.64%) and no occurrences in the 40 and 50 kg/m^2^ treatments (*p* = 0.04). There was also a tendency for the percentage of birds treated for aggressive damage to the head to be higher at both high and low SD (0.41, 0.31, 0, and 1.91% for treatments 30, 40, 50, and 60 kg/m^2^, respectively; *p* = 0.08, quadratic).

The incidence of aggressive damage (percentage of birds treated and/or culled) was also calculated for each 4-week period and showed no significant differences across treatments at 0–4, 8–12, and 12–16 weeks. From 4 to 8 weeks of age there was a quadratic relationship as SD increased, with birds at 30 and 60 kg/m^2^ experiencing the highest incidence of aggressive damage (3.28, 2.48, 2.27, and 8.90% for treatments 30, 40, 50, and 60 kg/m^2^, respectively; *p* = 0.04). Furthermore, the incidence of aggressive damage was evaluated by location of damage on treated birds only for each 4-week period (excludes birds culled for aggressive damage). At 0–4 weeks, there was a quadratic relationship with birds at the 60 kg/m^2^ treatment receiving the most damage to the tail area, followed by 30 kg/m^2^ (0.41, 0, 0.25, and 1.27% for treatments 30, 40, 50, and 60 kg/m^2^, respectively; *p* = 0.04). At 4–8 weeks, birds housed at 60 kg/m^2^ experienced more aggressive damage to the neck (0, 0, 0.51, and 0.85 for treatments 30, 40, 50, and 60 kg/m^2^, respectively; *p* = 0.04, linear) and to the snood (0, 0, 0, and 0.85 for treatments 30, 40, 50, and 60 kg/m^2^, respectively; *p* < 0.01, quadratic) compared to other densities. There were no differences for weeks 8–12.

### Heterophil/lymphocyte ratio

The heterophil/lymphocyte ratio of toms increased linearly as SD increased at week 4 (0.65, 0.77, 0.75, and 0.79 for treatments 30, 40, 50, and 60 kg/m^2^, respectively; *p* = 0.01). For week 12, there was a tendency for a linear relationship with increasing H/L ratios as SD increased (0.93, 0.89, 1.10, and 1.01 for treatments 30, 40, 50, and 60 kg/m^2^, respectively; *p*-value of 0.07). At 16 weeks of age, treatment had no impact on bird H/L ratio (0.86, 0.76, 0.85, and 0.90 for treatments 30, 40, 50, and 60 kg/m^2^, respectively).

### Behavioral observations

At 12 weeks of age (Table 6), SD impacted the percentage of toms standing and walking, as well as the incidence of total disturbances. The percentage of birds standing demonstrated a quadratic relationship with increasing SD (16.84, 15.04, 14.54, and 19.54 for treatments 30, 40, 50, and 60 kg/m^2^, respectively; *p* = 0.02). The percentage of birds walking decreased linearly with increasing SD, as more birds were observed walking at lower densities (*p* = 0.03). Additionally, the percentage of birds experiencing disturbances demonstrated a decreasing linear relationship with increasing density (*p* = 0.01).

**Table 6:** Effect of estimated final stocking density on percentage of turkey toms performing various behaviors (% of birds within the field of view) at 12 weeks of age.

**Behavior**	***n***	**Estimated final stocking density (kg/m**^**2**^**)**				**SEM^a^**	***p*-value (linear)**	***p*-value (quadratic)**	**Regression equation^b^**

		**30**	**40**	**50**	**60**				
Resting	2	60.56	66.06	67.53	63.18	1.980	0.54	0.32	–
Standing	2	16.84	15.04	14.54	19.54	1.063	0.10	0.02	Y = 0.02x^2^ − 1.45x+45.37
Walking	2	5.21	4.32	2.67	2.64	0.591	0.03	0.41	Y = −0.09x + 7.93
Feeding	2	6.48	5.00	5.35	5.55	0.684	0.33	0.58	–
Drinking	2	2.63	1.87	2.01	2.14	0.522	0.42	0.97	–
Preening	2	4.57	4.65	5.06	4.60	0.267	0.15	0.96	–
Comfort^c^	2	0.47	0.34	0.44	0.19	0.056	0.80	0.37	–
Environmental pecking	2	2.22	1.58	1.18	1.33	0.192	0.72	0.14	–
Feather pecking	2	0.40	0.72	0.73	0.50	0.085	0.29	0.16	–
Aggressive pecking	2	0.34	0.13	0.30	0.12	0.045	0.64	0.97	–
Dominance^d^	2	0	0.10	0.08	0.17	0.037	0.21	0.98	–
Total disturbance^e^	2	0.27	0.16	0.11	0.05	0.035	0.01	0.81	Y = −0.01x + 0.46

a *Standard error of the mean*.

b *Regression considered significant if p ≤ 0.05*.

c *Comfort: stretching, wing flapping, dustbathing, head scratching, and feather ruffling*.

d *Dominance: strutting, fighting, and posturing*.

e *Total disturbance: overall disturbances and severe disturbances*.

At 14 weeks of age, SD affected the percentage of birds seen resting, walking, preening, and performing comfort behaviors (Table 7). The percentage of birds resting increased with increasing SD with 53.98, 60.64, 68.21, and 64.34% of birds resting for 30, 40, 50, and 60 kg/m^2^, respectively (*p* = 0.02, linear; trend *p* = 0.07, quadratic). Walking behavior demonstrated a decreasing linear relationship with increasing SD (*p* = 0.04). The percentage of birds preening and performing comfort behaviors both increased linearly in relation to increasing SD (*p* = 0.01 and *p* = 0.03, respectively).

**Table 7:** Effect of estimated final stocking density on percentage of turkey toms performing various behaviors (% of birds within the field of view) at 14 weeks of age.

**Behavior**	***n***	**Estimated final stocking density (kg/m**^**2**^**)**				**SEM^a^**	***p*-value (linear)**	***p*-value (quadratic)**	**Regression equation^b^**

		**30**	**40**	**50**	**60**				
Resting	4	53.98	60.64	68.21	64.34	1.880	0.02	0.07	Y = 0.39x + 44.41
Standing	4	22.22	19.36	17.52	18.76	0.787	0.91	0.76	–
Walking	4	5.86	4.59	2.54	3.21	0.423	0.04	0.24	Y = −0.10x + 8.56
Feeding	4	7.55	5.15	2.63	4.09	0.590	0.09	0.08	–
Drinking	4	4.50	2.43	1.92	2.73	0.539	0.94	0.28	–
Preening	4	3.02	4.66	4.60	4.74	0.288	0.01	0.15	Y = 0.05x + 1.96
Comfort^c^	4	0.12	0.40	0.22	0.37	0.045	0.03	0.30	Y = 0.57e^−2^x + 0.02
Environmental pecking	4	1.44	1.53	1.18	0.99	0.154	0.90	0.42	–
Feather pecking	4	0.65	0.62	0.81	0.41	0.080	0.88	0.15	–
Aggressive pecking	4	0.34	0.23	0.09	0.11	0.044	0.06	0.58	–
Dominance^d^	4	0.32	0.15	0.11	0.12	0.038	0.07	0.45	–
Total disurbance^e^	4	0.05	0.17	0.15	0.18	0.030	0.06	0.60	–

a *Standard error of the mean*.

b *Regression considered significant if p ≤ 0.05*.

c *Comfort: stretching, wing flapping, dustbathing, head scratching, and feather ruffling*.

d *Dominance: strutting, fighting, and posturing*.

e *Total disturbance: overall disturbances and severe disturbances*.

At week 16, SD affected the percentage of birds resting, standing, walking, feeding, drinking, preening, and engaging in aggressive pecking (Table 8). The percentage of birds resting (45.88, 62.01, 68.14, and 59.05; *p* < 0.01) and standing (22.99, 18.12, 17.20, and 24.54; *p* = 0.01) demonstrated quadratic relationships as SD increased for treatments 30, 40, 50, and 60 kg/m^2^, respectively. Walking, feeding, and drinking behavior showed decreasing quadratic relationships with increasing SD (*p* = 0.02, *p* = 0.02, and *p* = 0.05, respectively). The percentage of birds that were preening increased linearly as SD increased (*p* = 0.02). Finally, the percentage of birds engaging in aggressive pecking increased linearly with increasing SD (*p* = 0.04).

**Table 8:** Effect of estimated final stocking density on percentage of turkey toms performing various behaviors (% of birds within the field of view) at 16 weeks of age.

**Behavior**	***n***	**Estimated final stocking density (kg/m**^**2**^**)**				**SEM^a^**	***p*-value (linear)**	***p*-value (quadratic)**	**Regression equation^b^**

		**30**	**40**	**50**	**60**				
Resting	4	45.88	62.01	68.14	59.05	2.509	0.01	< 0.01	Y = −0.06x^2^ + 6.11x − 81.23
Standing	4	22.99	18.12	17.20	24.54	1.330	0.22	0.01	Y = 0.03x^2^− 2.70x + 76.77
Walking	4	6.92	3.94	2.33	2.97	0.527	0.01	0.02	Y = 0.01x^2^ − 0.94x + 27.18
Feeding	4	9.06	4.11	2.78	3.36	0.784	0.03	0.02	Y = 0.01x^2^ − 1.43x + 39.49
Drinking	4	6.08	3.12	2.04	2.93	0.531	0.14	0.05	Y = 0.01x^2^− 0.97x + 26.58
Preening	4	2.73	3.82	4.08	4.02	0.363	0.02	0.34	Y = 0.04x + 1.72
Comfort^c^	4	0.34	0.45	0.46	0.19	0.077	0.94	0.17	–
Environmental pecking	4	2.54	1.81	1.37	0.83	0.302	0.30	0.55	–
Feather pecking	4	0.96	1.02	0.61	0.82	0.157	0.97	0.88	–
Aggressive pecking	4	0.20	0.10	0.14	0.27	0.043	0.04	0.20	Y = 0.19e^−2^x + 0.09
Dominance^d^	4	2.03	1.10	0.40	0.64	0.248	0.13	0.39	–
Total disturbance^e^	4	0.26	0.42	0.49	0.40	0.087	0.20	0.44	–

a *Standard error of the mean*.

b *Regression considered significant if p ≤ 0.05*.

c *Comfort: stretching, wing flapping, dustbathing, head scratching, and feather ruffling*.

d *Dominance: strutting, fighting, and posturing*.

e *Total disturbance: overall disturbances and severe disturbances*.

## Discussion and conclusions

While bird health and welfare are important, few studies have focused on bird wellbeing with respect to SD of heavy toms. Health and wellbeing of birds can be difficult to evaluate, as a result multiple parameters have been evaluated in combination in the current study to assess the effects of SD on turkey toms. The current stocking density recommendations in Canada vary based on bird body weight and additional environmental and management conditions. These recommendations must be met by the producer in order to house turkeys at the higher SD (50–60 kg/m^2^ for birds weighing 10.8–13.3 kg to 55–65 kg/m^2^ for birds weighing above 13.3 kg) (23). These target weights are important as they relate to the desired end use of the bird (whole bird vs. further processing) and therefore it is important to consider the impact of SD on birds marketed at both lighter and heavier weights.

Mobility is concerning from a bird health and welfare standpoint, with poor mobility impacting the bird's ability to access food and water, escape aggressive pen mates, and potentially resulting in pain (24–27). Poor mobility and decreased activity levels have also been related to poorer skeletal health in turkeys reared at high SD (28). Bird mobility (subjective gait score) demonstrated no differences at 12 weeks of age, while at week 16 bird mobility was significantly poorer with increasing SD. These findings are similar to Martrenchar et al. who observed that gait score was poorer in 12-week hens and 16-week toms housed at high SD (2). It has been hypothesized that the decrease in mobility seen with increasing SD may be a result of reductions in bird activity due to space (2). This is further supported by the changes seen in tom behavior, with greater space allowance at low SD allowing for more activity, as shown by the decreased percentage of birds walking with increasing SD (12, 14, and 16 weeks).

The increase in footpad lesions (week 16) may also relate to the decrease in mobility, as footpad lesions have been associated with pain (5, 6). Similar results have been seen in turkeys previously, with a higher incidence of footpad lesions present at higher SD in 12-week old hens and 16-week old toms at slaughter (2). Increases in the frequency of footpad lesions have been closely linked to increases in litter moisture (2, 5, 29, 30). Litter moisture has often been associated with increasing SD likely as a result of increased excreta output, but also due to reduced exposure of the litter to the air as it is more densely populated with birds (2, 31–33). Litter moisture in the current study demonstrated no significant difference across density treatments (34), suggesting there may be other factors relating to the increase in footpad lesions with increasing SD.

Standing and resting behavior may also be related to the increase in footpad lesions seen with increasing SD. Birds housed at moderate SD were seen resting more frequently, while footpad lesions increased linearly with increasing SD. Since the birds housed at 50 kg/m^2^ showed a higher percentage of birds resting, it could be suggested that birds housed at 60 kg/m^2^ did not have the space to rest comfortably and a higher percentage of birds are seen standing as a result. In addition, the percentage of birds being disturbed showed a linear decrease with increasing SD (12 weeks), which may be a result of the improvement in mobility as more birds are moving around at low SD (30 kg/m^2^). Broiler chicken research has shown similar results, with decreases in activity in relation to higher SD (33, 35). However, these results contradict the previous research in turkeys in which there was a tendency for more disturbances at higher housing densities (2). It is important to note that the densities in the previous study were lower than the SD evaluated in the current study.

Birds that have difficulty walking may take longer to reach feed and water or avoid getting up to access feed, resulting in reductions in body weight and in severe instances dehydration and culls (27). This is further supported by the decrease in feeding and drinking behavior in older birds (16 weeks) as well as the decrease in final body weights seen as SD increases (36), as poor mobility may impact the bird's ability to access resources such as feed and water. In this study, the reduction in nutritive behaviors is not a result of available feeder and drinker space, as it was allocated on a per bird basis. It is therefore likely a result of reduced mobility, social stress, or increased difficulty getting to the feeders and drinkers. This has been suggested previously by Simitzis et al. where the authors noted that birds became less active and had more difficulty walking past pen mates often bumping into each other at higher SD (37).

Other factors that may result in decreased performance are increased stress and immune function. Few studies have evaluated the H/L ratio in relation to turkey SD, however one study found no effect with increasing SD (up to 58 kg/m^2^) in turkey toms from 7, 12, 16, and 20 weeks of age (3). This could suggest that either SD or group number impacts stress levels at younger ages, rather than later in life. In the current study, the early increase in the H/L ratio (week 4) in relation to increasing SD indicates that young birds may be experiencing stress due to increased SD or increased group size. To the authors' knowledge, group size has not been thoroughly evaluated for young turkeys or in group sizes larger than 100 turkeys, however previous studies suggest that increasing group size has been associated with increased stress levels and aggression in both older laying hens and turkeys (38–40). The early H/L increase may also relate to the decrease in feed efficiency early on (week 4–8). During the stress response, corticosterone release results in a redirection of energy (glucose) stores, whereby growth in the form of protein accretion is slowed as glucose utilization is restricted (41). The body initially directs energy toward the adaptive immune response, resulting in stimulation of antibodies, stimulation of heterophil production, and reduction in lymphocyte numbers (41). In the event of a chronic stressor, energy is directed toward maintaining the stress response and less energy is available for the innate immune system and growth (41–43) resulting in a greater energy expenditure and may lead to poorer feed efficiency. This is further supported as the H/L ratio at 12 weeks of age followed the same trend seen at 4 weeks, suggesting that birds are still experiencing some form of stress due to increased SD resulting in poorer feed efficiency and lower final body weights. Other behavioral changes may be indicative of stress, which could also help explain the poorer feed efficiency throughout the study at high SD. The increased SD as a result of increasing group size may relate to higher levels of aggression at high densities, as seen by an increase in aggressive pecking behavior in older birds (week 16).

Aggressive damage is a major welfare concern in turkeys as it is painful and can lead to unnecessary culling of otherwise healthy birds (44). In this study, aggressive damage included any bird that demonstrated an open lesion as a result of pecking or as a result of a skin tear. Skin tears were included, as they typically attract attention of pen mates resulting in further bird damage due to pecking. The total incidence of aggressive damage separated by age demonstrated a quadratic relationship with increasing SD in young birds 4–8 weeks, highest in both 30 and 60 kg/m^2^); to the authors' knowledge, this has not previously been seen in young poults in relation to SD. A previous study evaluating the effects of SD on aggressive behavior demonstrated no effects in birds as young as 6 weeks of age (2). In addition, previous studies have seen increases in aggression with decreases in space allowance in older birds, which was not seen in the current study (44). Denbow et al. also evaluated behavior at 12 and 20 weeks in relation to SD and found no effects, however they did not evaluate antagonistic behavior in younger birds (4). When evaluated by the location of aggressive damage, young birds (0–4 and 4–8 weeks) supported an increase in aggressive damage with increasing SD that was seen in older birds (44), with more damage seen on the tail, neck, and snood. The current study also found a quadratic effect on the incidence of skin tears, with damage occurring at both extremes (30 and 60 kg/m^2^). Although not necessarily a result of aggression, the increase in skin tears may be the result of increased activity at low SD (increased percentage of birds walking and decreased percentage of birds resting) as well as decreased space for birds to walk past resting birds at high SD. This may be further supported by the decrease in back feather condition with increasing SD at older ages (week 12 and 16) possibly in relation to minimal bird space. Although aggressive damage was not significantly different in older birds, behavioral observations indicate that aggressive pecking occurs more frequently at both low and high density as opposed to moderate densities. This could occur for two reasons, birds at low SD may be more active and as a result may participate in more aggressive encounters, while it is also possible that birds at high SD are exhibiting frustration consistent with the increase in preening.

Poor feather condition may indicate damaged feathers or missing feathers due to feather pecking or increased wear. The removal of feathers has been associated with pain (45), indicating that more aggressive forms of feather pecking may be detrimental to bird health and wellbeing. Overall feather condition was consistently poorer at high SD for toms at 10, 12, and 16 weeks of age. Although feather condition is not frequently evaluated in relation to turkey SD, these findings are consistent with those seen in 14-week old turkeys housed at high SD, where poorer feather condition was seen with increasing density (1). The back feathers were more severely impacted in older birds (week 12 and 16), likely when toms become limited by space and may walk over top of other birds. Tail feather condition consistently decreased with increased SD which may be due to space restrictions developing as birds grow, with tail feathers being the most severely impacted as they likely experience more friction as birds are moving past each other at higher SD. The tail feathers are also likely to be stepped on more frequently as birds are resting due to the space restrictions seen with increasing SD. Another factor impacting tail condition could be feather pecking, although not seen statistically, the incidence birds treated for aggressive damage to the tail occurred at the highest frequency in the 60 kg/m^2^ treatment. Wing feather condition may also be impacted by friction due to increased bird contact at older ages (week 16). The breast feather score was poorer at high SD (week 10, 12, and 16) and although turkeys do not typically have a lot of feathering on the breast, poorer feathering at high densities is likely attributed to increased contact with wet litter (46). A study conducted with laying hens demonstrated similar effects with increasing density resulting in decreases in feather condition for the neck, chest, tail, back, and wings (19). The authors suggested that the hens at low densities did not exhibit as much feather pecking as they had a greater space allowance. This may not be the case in the current study, as there was no impact of SD on aggressive damage at older ages.

Feather cleanliness may also be related to bird health and performance. The decrease in feather cleanliness may be related to decreases in litter quality, which has been associated with increasing SD in previous studies (2, 31). Although litter moisture was not higher in the current study (34), it could be suggested that the increased fecal output per unit of space would contribute to the decrease in cleanliness. Poorer feather condition and cleanliness may also relate to decreases in core body temperature, as feathers that come into contact with excreta from the litter become wet and dirty and birds are more likely to lose heat to the environment (47). As a result, more of the bird's resources may have been directed toward maintenance or thermoregulation rather than growth, which has been suggested in previous literature (20, 48). This is further supported in the current study with decreases in feed efficiency as SD increases (36). Behavioral changes also support poorer feather condition and cleanliness with increasing levels of SD, with more birds preening at higher SD. While preening behavior is often associated with either comfort behavior or displacement behavior, it also has a functional component for maintaining and cleaning plumage (49, 50). It could be suggested that the increase in preening behavior could be associated with decreases in feather cleanliness seen as SD is increased.

In conclusion, high SD significantly impacts turkey tom health and welfare to 16 weeks of age. It was hypothesized that high SD would result in increased competition and aggression, resulting in stress. There was no impact of SD on the H/L ratio at older ages, however other parameters measured suggest that the birds are likely experiencing stress in relation to increasing SD. High SD (60 kg/m^2^) resulted in poorer mobility, increased footpad lesions, poor feather condition, and decreased feather cleanliness. Despite the concerns of higher SD, these data also indicate that very low SD (30 kg/m^2^) in enclosed spaces may be less than ideal with regards to particular variables. Aggressive pecking behavior (week 16) was higher at low SD, compared with moderate densities (40 and 50 kg/m^2^). The percentage of birds disturbed while resting was highest at low SD compared with all other densities at 12 weeks of age, and the incidence of skin tears was also higher at low SD (weeks 12–16), with no skin tears seen at moderate densities. All of these factors could potentially relate to the levels of increased activity that were seen with turkeys housed at low SD. Birds that are more active, such as those at low SD, may be more likely to be involved in aggressive encounters or injure other pen mates. As a result very low housing densities, while suitable for performance (36), may not always favor bird health and wellbeing. To achieve optimal bird health and wellbeing low to moderate densities may be ideal, however performance parameters should also contribute when selecting optimal SD.

## Ethics statement

This study was carried out in accordance with the recommendations of the Guide to the care and use of experimental animals and the CCAC guidelines on: the care and use of farm animals in research, teaching and testing, Canadian Council on Animal Care (51, 52). The protocol was approved by the University of Saskatchewan's Animal Care Committee.

## Author contributions

KS-L was the primary investigator and KB collected, analyzed, and interpreted data, and drafted the manuscript. Both authors approved the final manuscript.

### Conflict of interest statement

The authors declare that the research was conducted in the absence of any commercial or financial relationships that could be construed as a potential conflict of interest.
